# Do we need patient‐specific QA for adaptively generated plans? Retrospective evaluation of delivered online adaptive treatment plans on Varian Ethos

**DOI:** 10.1002/acm2.13876

**Published:** 2022-12-22

**Authors:** Xiaodong Zhao, Dennis N. Stanley, Carlos E. Cardenas, Joseph Harms, Richard A. Popple

**Affiliations:** ^1^ Department of Radiation Oncology Washington University in St. Louis St. Louis Missouri USA; ^2^ Department of Radiation Oncology University of Alabama at Birmingham Birmingham Alabama USA

**Keywords:** online adaptive radiotherapy, patient‐specific quality assurance, second check dosimetry

## Abstract

**Background:**

The clinical introduction of dedicated treatment units for online adaptive radiation therapy (OART) has led to widespread adoption of daily adaptive radiotherapy. OART allows for rapid generation of treatment plans using daily patient anatomy, potentially leading to reduction of treatment margins and increased normal tissue sparing. However, the OART workflow does not allow for measurement of patient‐specific quality assurance (PSQA) during treatment delivery sessions and instead relies on secondary dose calculations for verification of adapted plans. It remains unknown if independent dose verification is a sufficient surrogate for PSQA measurements.

**Purpose:**

To evaluate the plan quality of previously treated adaptive plans through multiple standard PSQA measurements.

**Methods:**

This IRB‐approved retrospective study included sixteen patients previously treated with OART at our institution. PSQA measurements were performed for each patient's scheduled and adaptive plans: five adaptive plans were randomly selected to perform ion chamber measurements and two adaptive plans were randomly selected for ArcCHECK measurements. The same ArcCHECK 3D dose distribution was also sent to Mobius3D to evaluate the second‐check dosimetry system.

**Results:**

All (*n* = 96) ion chamber measurements agreed with the planned dose within 3% with a mean of 1.4% (± 0.7%). All (*n* = 48) plans passed ArcCHECK measurements using a 95% gamma passing threshold and 3%/2 mm criteria with a mean of 99.1% (± 0.7%). All (*n* = 48) plans passed Mobius3D second‐check performed with 95% gamma passing threshold and 5%/3 mm criteria with a mean of 99.0% (± 0.2%).

**Conclusion:**

Plan measurement for PSQA may not be necessary for every online‐adaptive treatment verification. We recommend the establishment of a periodic PSQA check to better understand trends in passing rates for delivered adaptive treatments.

## 1 INTRODUCTION

Adaptive radiotherapy has been increasingly implemented clinically due to its ability to create treatment plans that account for daily anatomical changes of both target and surrounding organ‐at‐risk (OAR).^1^, ^2^ Commercial platforms are available that provide either MRI‐guided ART (MRgART)^3^, ^4^, ^5^ or CT (or cone‐beam CT)‐guided ART (CTgART).^6^, ^7^, ^8^ Varian Ethos (Varian Medical Systems, Inc., Palo Alto, CA) is a CTgART platform that allows for online adaptive radiation therapy (OART) utilizing daily kV‐CBCT.^6^, ^7^, ^8^

The Ethos treatment unit incorporates 4 main components for OART: CBCT on‐board imaging, a 6 MV flattening‐filter‐free beam treatment delivery system, an adaptive treatment planning workspace, and an independent plan QA system, Mobius3D‐Adapt (Varian Medical Systems, Inc., Palo Alto, CA). Using these components, the Ethos system is able to efficiently generate daily online‐adapted treatment plans with minimal human interaction.^9^ In daily online adaptive RT, there are 3 different plans that could be evaluated. They are the following: (1) A reference plan, planned on the simulation CT, that is created prior to treatment. (2) On the day of treatment, a CBCT is acquired, and structures are drawn on the anatomy of the day. Using these structures, a structure‐guided deformable registration is applied, and a synthetic CT (sCT) is generated. The scheduled plan is generated on the sCT and displayed on CBCT for user evaluation and review. The scheduled plan represents the treatment plan as initially optimized and re‐calculated on the approved contour and the sCT of the session. (3) The adapted plan represents a newly optimized plan based on the same contours and sCT used for the generation of the scheduled plan.

The importance of an integrated quality assurance (QA) software system has been previously discussed in the literature.^10^, ^11^ Currently, the standard practice is to validate the plan with a secondary dosimetry system and with patient‐specific QA measurements. Examples of vendor‐provided solutions include independent Monte Carlo dose computations^12^, ^13^ or collapsed‐cone‐based dose calculations.^14^ However, these secondary dose calculation software are usually provided by the same vendor as the original treatment planning system (TPS), as is the case for Ethos and Mobius3D. In‐house software has been used to show that real‐time electronic portal imaging detector (EPID) dosimetry could be used to detect errors.^15^ Post‐treatment log‐file‐based solution has also been employed clinically and are commercially available.^16^, ^17^, ^18^, ^19^ Transmission detectors are also under development by multiple groups for real‐time dosimetric monitoring.^20^, ^21^ Other QA tools also compare important plan parameters such as beam angles, segment fluence patterns, and beam‐on time between the previously approved plan to the re‐optimized plan. Physicists would review these differences and approve the plan if reasonable.^10^ The current adapted planning workflow for the Ethos system includes secondary dose calculation of the adaptive plans but lacks a mechanism to perform PSQA prior to delivery of the selected treatment plans due to the unique nature of the plan creation with the patient in the treatment position.

With the unique QA challenges of an expedited treatment time frame and the need to reduce patient on‐table time, adaptive treatment deliveries pose increased risks that are not typically considered for non‐adaptive treatments.^11^ Therefore, there is a need to conduct a comprehensive analysis of delivered adapted plans’ quality through PSQA measurements. This study aims to quantify the PSQA results of adaptive plans previously treated with online adaptive therapy to increase the medical physics community's confidence in the current workflow.

## 2 MATERIALS AND METHODS

Sixteen patients previously treated at our institution with online adaptive radiotherapy using the Ethos treatment delivery system were selected for this IRB‐approved study. Patients were randomly selected to include a diverse sample of treatment sites and modalities, as shown in Table 1. Patient PTV volume ranges from 72.20 to 1453.01 cc, with a mean of 519.07 cc. Patients were treated with intensity‐modulated radiotherapy (IMRT) plans using 6–10 fields (*n* = 14) or volumetric modulated arc therapy (VMAT) plans using 2–3 arcs (*n* = 2). The selected distribution of IMRT and VMAT plans in the study was consistent with the distribution of plan modalities used in the clinic.

**TABLE 1:** Summary of treatment sites and modalities

Patient Number	Site	Dose/fractionation	Plan type	Initial PTV size (cc)
1	Prostate	Conventional fractionation	IMRT	372.57
2	Mediastinum	Conventional fractionation	IMRT	298.57
3	Other abdomen	Conventional fractionation	VMAT	214.11
4	Cervix	Conventional fractionation	IMRT	1321.65
5	Prostate	Conventional fractionation	IMRT	942.74
6	Rectum	SBRT	IMRT	92.55
7	Bladder	Conventional fractionation	IMRT	207.18
8	Prostate	Conventional fractionation	IMRT	1021.47
9	Left breast	SBRT	IMRT	82.75
10	Mediastinum	Conventional fractionation	IMRT	317.26
11	Retroperitoneal space	Conventional fractionation	VMAT	249.84
12	Prostate	Conventional fractionation	IMRT	1229.33
13	Prostate	Conventional fractionation	IMRT	144.69
14	Cervix	Conventional fractionation	IMRT	1453.01
15	Prostate	Conventional fractionation	IMRT	285.16
16	Right breast	SBRT	IMRT	72.20

**FIGURE 1:**
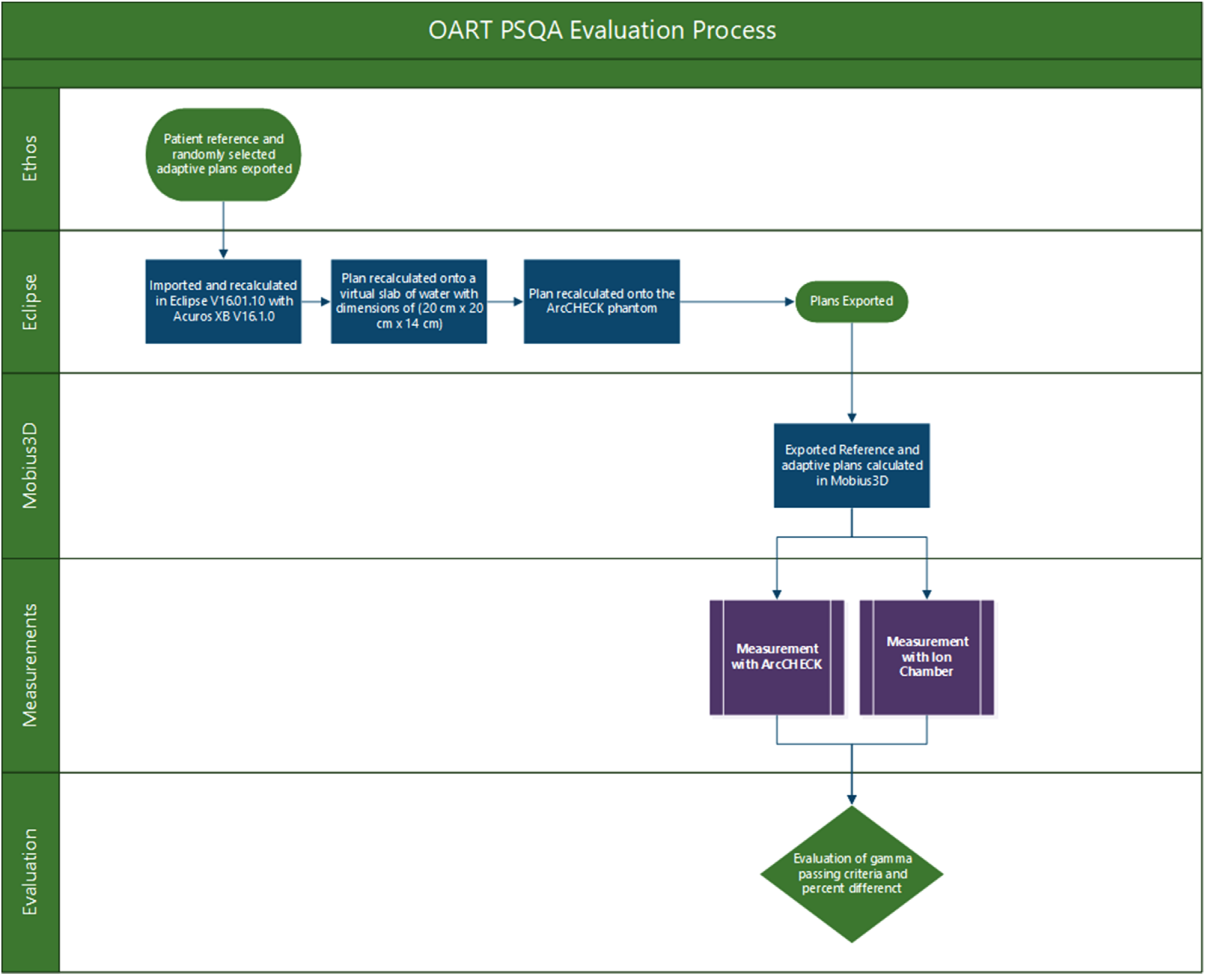
Flowchart of OART PSQA evaluation process

One scheduled plan and five randomly selected adaptive plans were chosen for each patient and ion chamber measurements were performed for these plans, where the scheduled plan serves as a reference. As mentioned in the introduction, the scheduled plan represents the treatment plan as initially optimized and re‐calculated on the approved contour and the sCT of the session, where sCT is the planning CT deformed to the CBCT prior to treatment. The adapted plan represents a newly optimized plan based on the same contour and sCT. The scheduled plan represents the initial fluence which was measured at pre‐treatment PSQA. The PSQA measurement in this study does not take online deformation into account since it is calculated on the phantom. A 0.125 cm^3^ ion chamber (PTW N31010 PTW Dosimetry, Freiburg, Germany) embedded in the center of 20cm×20cm×14cm solid water phantom was utilized for measurement. The position of the solid water was chosen for the ion chamber to be within a high‐dose region without sharp dose gradients. The ion chamber volume is modeled in TPS with an air cylinder of 0.125 cc embedded in the middle of the water phantom with the same dimension as the solid water phantom. The average expected dose over the volume of the air cylinder is reported compared to the measurement. Subsequently, the scheduled plan and two of the selected adaptive plans were delivered to a cylindrical diode array (ArcCHECK, Sun Nuclear, Melbourne, FL) and analyzed using SNC Patient software (Sun Nuclear, Melbourne, FL). SNC Patient software converts the 3D calculated dose of the phantom into an expected 2D diode array reading and subsequently compared it to the measured 2D diode array data by using gamma analysis. Both ion chamber and ArcCHECK expected dose were calculated in Eclipse version 16.01.10 with Acuros XB version 16.1.0 (Varian Medical Systems, Inc., Palo Alto, CA) with a dose grid resolution of 2.5mm×2.5mm×2.5mm. The ArcCHECK 3D dose distributions were also sent to the secondary dose calculation software Mobius3D for evaluation on the secondary dose calculation accuracy, where the Mobius3D calculated 3D dose is compared with TPS calculated 3D dose based on gamma analysis. The process was summarized in the flowchart in Figure 1.

Passing criteria for PSQA followed departmental procedure: 3% difference between Eclipse expected dose to measured dose for ion chamber measurement, and 95% gamma^22^, ^23^ threshold using 3%/2 mm criteria with excluding 10% low dose region for ArcCHECK, and 95% gamma threshold using 5%/3 mm criteria for Mobius3D. The passing criteria for Mobius3D were chosen based on recommendations from Task Group 219 on independent calculation‐based dose/MU verification for IMRT and our institutional clinical experience.^24^ Additionally, the Mobius3D dosimetric leaf gap (DLG) was evaluated using the vendor's recommended procedure. For the sake of this study, no adjustment to the vendor‐provided DLG (0 mm) is made.

## 3 RESULTS

**FIGURE 2:**
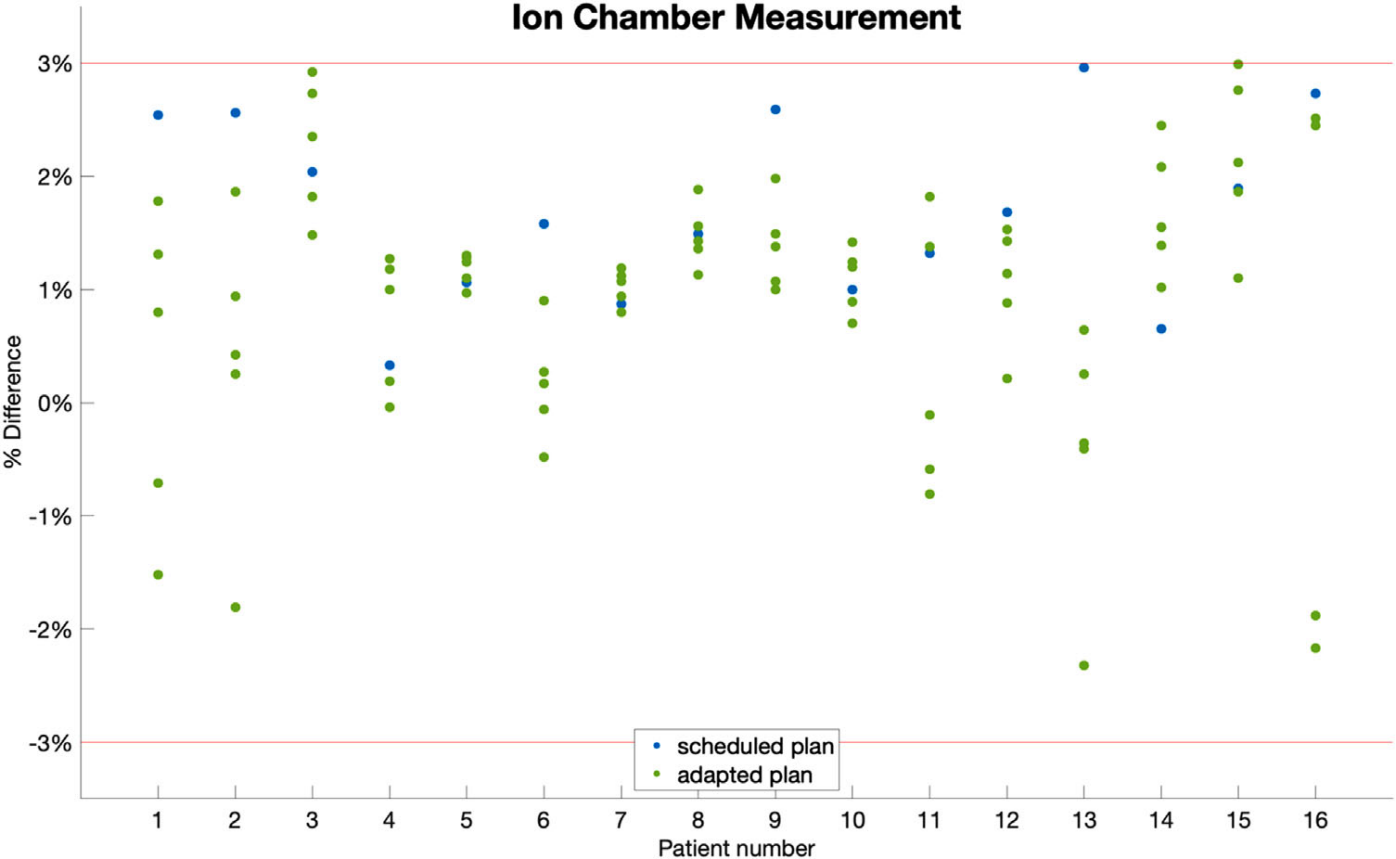
Difference between planned and delivered dose for all plans as measured with an ion chamber. The blue dots represent scheduled plans and the green dots represent adapted plans. The red line indicates the passing threshold

Ion chamber measurement results are shown in Figure 2. All ion chamber measured doses are within 3% of the expected dose in Eclipse. The maximum ion chamber deviation is 2.9% and the mean ion chamber deviation is 1.4% (± 0.7%). The scheduled plans’ mean ion chamber deviation is 1.7% (± 0.8%). The adapted plans’ mean ion chamber deviation is 1.3% (± 0.7%). The systematic offset between the calculation and ion chamber measurements can be contributed to the difference in dose calculation medium between Eclipse AcurosXB and Ethos AcurosXB systems, where Eclipse is set as dose to water versus Ethos which is dose to medium.

**FIGURE 3:**
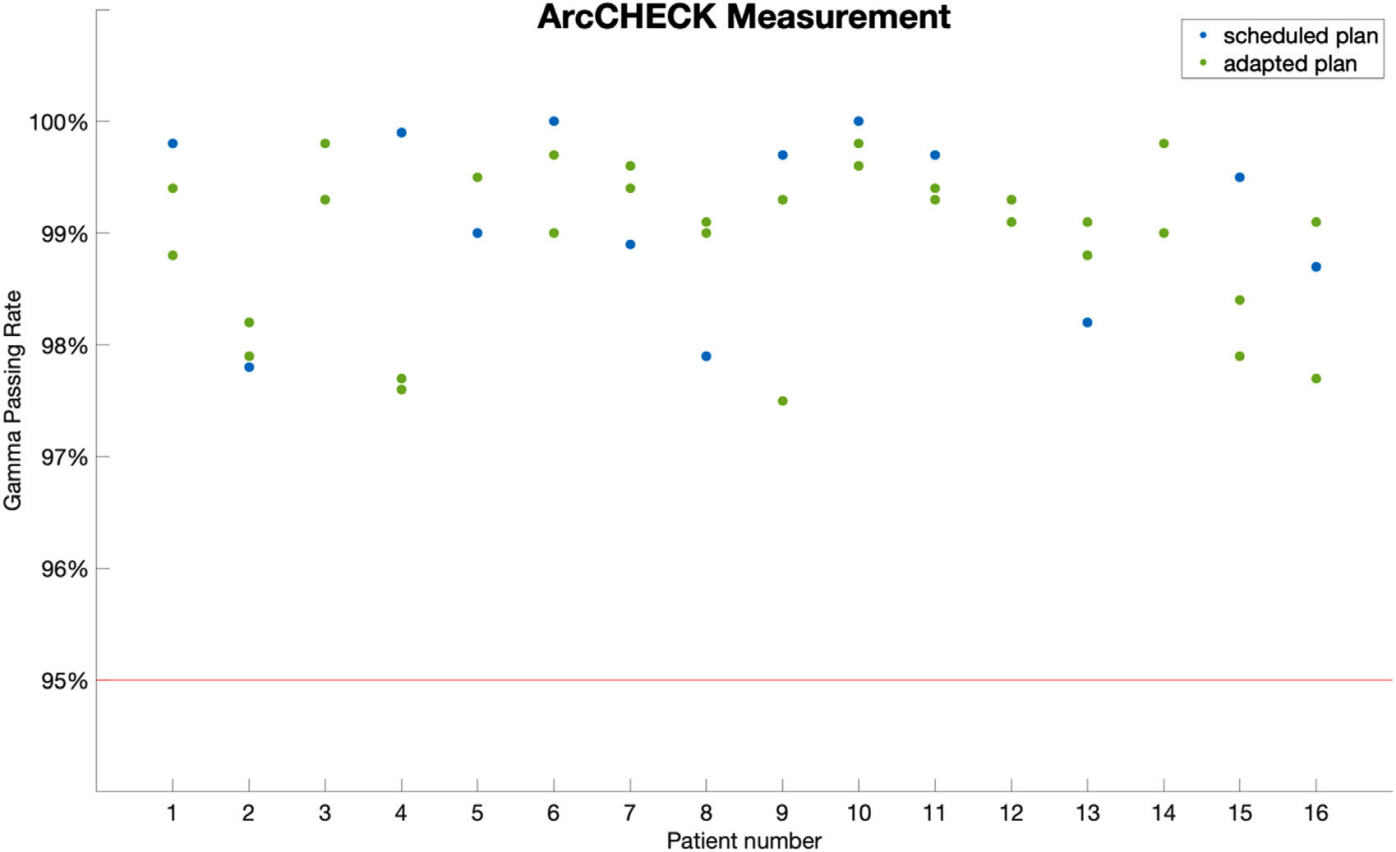
Gamma passing rate for ArcCHECK expected dose as the reference compared to the measurement for all plans. The blue dots represent scheduled plans and the green dots represent adapted plans. The red line indicates the passing threshold

ArcCHECK measurement results are shown in Figure 3 where the expected dose from the TPS was used as the reference distribution. All ArcCHECK measurements met the passing criteria. The minimum passing rate for all plans was 97.5% with a mean passing gamma rate of 99.1% (± 0.7%). The scheduled plans’ mean ArcCHECK gamma passing rate is 99.2% (± 0.8%). The adapted plans’ mean ArcCHECK gamma passing rate is 99.0% (± 0.7%).

**FIGURE 4:**
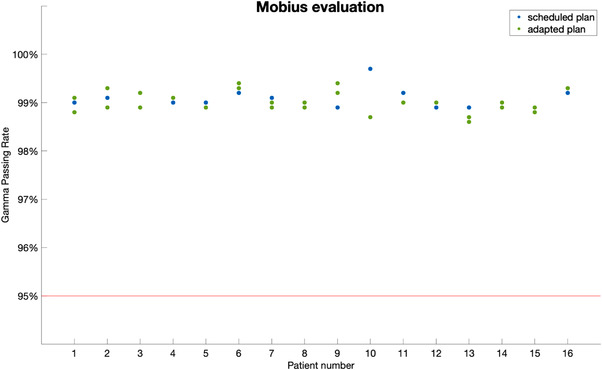
Gamma passing rate for the secondary dose calculation using Mobius compared to TPS calculated dose for all plans. The blue dots represent scheduled plans and the green dots represent adapted plans. The red line indicates the passing threshold

Mobius3D evaluation results are shown in Figure 4 where the TPS calculated dose was used as the reference. All Mobius3D secondary checks passed the 95% gamma threshold using 5%/3 mm criteria. The min passing rate of all plans is 98.6% with a mean of 99.0% (± 0.2%). The scheduled plans’ mean Mobius3D gamma passing rate is 99.1% (± 0.2%). The adapted plans’ mean Mobius3D gamma passing rate is 99.0% (± 0.2%). For this study, the order of the verification logic is as follows: (1) The measured data, which is the ground truth; (2) TPS verified against measurement; and (3) Mobius3D is verified against TPS.

## 4 DISCUSSION

Our study results show that Ethos OART treatment delivery without prior PSQA of adaptive (i.e., new) plans is within our institutional guidelines and recommendations of the American Association of Physicists in Medicine Task Group 218.^25^

Whether PSQA is necessary for quality control remains a controversial topic. It has been shown that PSQA is one of the least useful tools in effective error‐detection of commonly used quality control measures^26^ but is still considered a vital part of overall QA. Some studies have shown the efficiency and effectiveness of independent calculation software tools in the place of conventional measurement‐based PSQA as current treatment machines become increasingly stable.^17^, ^18^, ^27^ Surveys on PSQA practice patterns have also shown that some clinics rely on analysis of the delivery log in place of PSQA measurements.^28^, ^29^ Furthermore, there are also tools under development of predicting PSQA failure with artificial intelligence (AI).^30^, ^31^, ^32^, ^33^, ^34^

Current PSQA also has limitations. For example, most PSQA measurements are not truly 3D and are able to verify dose in only 2D or 1D. PSQA are also typically modeled as homogeneous materials which do not account for heterogeneity and patient‐specific anatomy in the measurement. Furthermore, setup uncertainties when aligning an independent detector for measurement, can affect the measurement accuracy. It is also hard to interpret the gamma passing rate in the context of clinical impact. For example, a pass rate below 95% does not necessarily mean that target coverage or OAR sparing will be compromised. In addition, in most clinics, the most common approach to a failing QA is to re‐measure multiple times or use an additional, less sensitive method of evaluation, which begs the necessity of PSQA.^29^, ^35^ Typically, ion chamber measurements are usually the last measure of PSQA quality in most clinics and are considered a key factor in determining if treatment should be approved. Additionally, in most QA validity studies, ion chamber measurements are used as a benchmark to determine the quality of the assessed QA tool. Independent measurement of the dose distribution does have some advantages over other verification methods.

In the meanwhile, PSQA measurements also have advantages. Measurements can capture errors where the TPS has limitations.^36^, ^37^ For example, if the TPS is configured for large fields, it might not model small fields very well. Plans with high modulation and small field openings might pass secondary dose calculation checks, but measurement would show a large discrepancy. Therefore, periodic measurement and review of PSQA for treated plans is an important tool for process control and continuous quality improvement.

Since OART is still a relatively new treatment technique, we recommend clinics establish periodic PSQA process control to retrospectively evaluate OART plans. Here, delivered plans could be sampled to document trends on adapted plans’ PSQA and maintain confidence in adaptively generated plan quality. This process should be repeated every couple of months for temporal consistency and verification, in addition to verifying any PSQA trend changes after any major software upgrades from individual vendors. Future studies will help determine the need to continue such quality control program efforts.

## 5 CONCLUSION

This study quantified the PSQA results of adaptive plans previously treated with online adaptive therapy to increase confidence in the current OART workflow. The data presented here has shown it is relatively safe to rely on independent secondary dose calculations for verification of Ethos OART plans. Additionally, PSQA may not be necessary for every adaptive treatment verification. We recommend the establishment of a periodic PSQA check to understand trends in passing rates for delivered adaptive treatments.

## AUTHOR CONTRIBUTIONS

Xiaodong Zhao made substantial contributions to the conception and design of the work; the acquisition, analysis, and interpretation of data for the work; drafting the work and revising it critically for important intellectual content; gave final approval of the version to be published; and agrees to be accountable for all aspects of the work in ensuring that questions related to the accuracy or integrity of any part of the work are appropriately investigated and resolved. Dennis Stanley made substantial contributions to the conception and design of the work; the acquisition, analysis, and interpretation of data for the work; drafting the work and revising it critically for important intellectual content; gave final approval of the version to be published; and agrees to be accountable for all aspects of the work in ensuring that questions related to the accuracy or integrity of any part of the work are appropriately investigated and resolved. Carlos E. Cardenas made substantial contributions to the conception and design of the work; the acquisition, analysis, and interpretation of data for the work; drafting the work and revising it critically for important intellectual content; gave final approval of the version to be published; and agrees to be accountable for all aspects of the work in ensuring that questions related to the accuracy or integrity of any part of the work are appropriately investigated and resolved. Joseph Harms made substantial contributions to the conception and design of the work; the acquisition, analysis, and interpretation of data for the work; drafting the work and revising it critically for important intellectual content; gave final approval of the version to be published; and agrees to be accountable for all aspects of the work in ensuring that questions related to the accuracy or integrity of any part of the work are appropriately investigated and resolved. Richard A. Popple made substantial contributions to the conception and design of the work; the acquisition, analysis, and interpretation of data for the work; drafting the work and revising it critically for important intellectual content; gave final approval of the version to be published; and agrees to be accountable for all aspects of the work in ensuring that questions related to the accuracy or integrity of any part of the work are appropriately investigated and resolved.

## CONFLICT OF INTEREST

The authors declare that there is no conflict of interest.
